# High Frequency Loss of 17q11.2 and Downregulation of the Cancer Metastasis Suppression microRNA miR-193a-3p in Prostate Cancer Bone Metastasis

**DOI:** 10.3390/cancers18030403

**Published:** 2026-01-27

**Authors:** Elzbieta Stankiewicz, Sarah C. McCarley, Xueying Mao, Sakunthala Kudahetti, Tim Oliver, Jonathan Shamash, Trevor Graham, Daniel M. Berney, Yong-Jie Lu

**Affiliations:** Barts Cancer Institute, Queen Mary University of London, Charterhouse Square, London EC1M 6BQ, UK; e.stankiewicz@gumed.edu.pl (E.S.); s.mccarley@qmul.ac.uk (S.C.M.); mxyecho@hotmail.com (X.M.); s.kudahetti@qmul.ac.uk (S.K.); r.t.oliver@qmul.ac.uk (T.O.); jonathan.shamash2@nhs.net (J.S.); trevor.graham@icr.ac.uk (T.G.); daniel.berney@nhs.net (D.M.B.)

**Keywords:** prostate cancer, bone metastasis, genome-wide copy number change profile, 17q11.2 loss, miR-193a-3p, cyclin D1, uPA, cell invasion

## Abstract

**Background/Objectives:** Although 90% of prostate cancer (PCa) metastasis occurs in the bone, there are limited studies and rarely available genome-wide profiles at individual sample level for genomic copy number changes in the literature. **Methods:** We performed Affymetrix SNP 6.0 high-density microarray analysis to generate the genome-wide copy number change profiles for six cases of PCa bone metastases. A common genomic loss was confirmed by fluorescence in situ hybridization (FISH) in paraffin-embedded PCa bone metastasis samples together with primary PCa and benign prostate hyperplasia samples. We overexpressed the candidate miRNA in PCa cell lines and knocked down its target genes by siRNA transfection and investigated the effect on protein expression and cell viability, migration, and invasion abilities, respectively. Protein expression in PCa tissues was analyzed by immunohistochemical staining. **Results:** We provided high-resolution PCa bone metastasis profiles of six cases and identified potential bone metastasis-specific common genomic alterations, including a 1.6 mb region on 17q11.2, as well as those shared by non-bone metastatic PCa. The common 17q11.2 loss was confirmed by FISH in further 14/21 PCa bone metastasis samples but was only found in 9/151 primary PCa samples. The well-established tumor-suppressing miRNA located within this small genomic region, miR-193a-3p, was downregulated in both bone metastasis and primary PCa cases, leading to overexpression of cyclin D1 and uPA to promote cancer cell migration and invasion. Cyclin D1 was highly expressed in both localized PCa and bone metastasis samples, and the expression was significantly higher in the latter group (*p* = 0.013). **Conclusions:** We generated high-resolution copy number change profiles for bone metastasis samples. This led to the identification of a common, small genomic loss and downregulation of miR-193a-3p, which suppresses PCa bone metastasis through inhibition of its target proteins, providing new insight into bone metastasis development.

## Simple Summary

Metastasis is the main cause of death from prostate cancer (PCa), a common malignant disease. A total of 90% of PCa metastases occur in bone, and thus understanding the genetic changes underlying bone preference is critical. However, due to challenges in obtaining bone samples, limited bone metastasis-specific genomic alteration profiles, particularly at individual sample level, have been published. We generated individual genomic change profiles from six PCa bone metastasis samples and identified bone metastasis-specific genomic changes. One of them, loss of the 17q11.2 genomic region, was further confirmed by another method in further 14/21 PCa bone metastasis samples but only in 9/151 primary PCa. The function of a well-established tumor suppressor, miR-193a-3p, located in this lost genomic region, was reduced in bone metastasis, leading to overaction of two well-established genes to promote cancer metastasis. This paper provides new insight into bone metastasis development.

## 1. Introduction

Prostate cancer (PCa) is the most common male cancer in Western countries and both its incidence and mortality (in certain countries) in Africa, Asia, and Latin America/Caribbean are experiencing a significant increase [1,2]. More than 1,460,000 cases and 396,000 deaths globally had been estimated in 2022 [3]. Although many early-stage PCas are indolent and are not life-threatening, metastatic disease is still incurable, despite the development of effective therapies for advanced PCa treatment [4]. Further investigation of genetic drivers underlying metastatic PCa is still critical to improve the survival rate of PCa patients.

Bone is the preferred organ for PCa metastasis, being involved in 90% of metastatic PCa cases [5,6]. Furthermore, unlike other cancers, PCa bone metastasis lesions are usually associated with osteosclerotic instead of osteolytic bone deposits [7,8]. While Paget’s “seed and soil theory” theorizes that underlying mechanisms and the unique pathological feature of common PCa bone metastases [6,7,8], the specific genomic alterations in the cancer cell “seeds”, which confer advantage for dissemination from the primary tumor and adaptation to the new metastatic microenvironment, are not well established. These genetic alterations may only exist in a small subset of primary tumor cells or can be acquired during the metastasis process where multiple steps of invasion and survival selection occur. Identifying the genomic changes in bone metastasis using cancer samples from the bone is critical for understanding the molecular mechanisms underlying the bone preference of PCa metastases. As bone biopsy is very rarely undertaken in metastatic PCa, limited studies have been performed to investigate the genetic and molecular alterations in PCa bone metastasis [9,10,11,12,13,14,15,16]. This is in contrast to the vast amount of data on primary and non-bone metastasis tissues, mostly lymph node metastasis [9,10,11,12,14,17,18,19,20,21,22,23]. Examining the studies that used bone metastasis samples, they either reported the data from bone together with other organ metastasis without a separate bone metastasis sample profile [9,11,12] or just reported certain common genomic regions or genetic features, without providing genome-wide alteration profiles [13,14,15,16]. Therefore, although bone is the most common place for PCa metastasis, the genomic alteration pattern for PCa bone metastasis is still unclear.

To identify genomic alterations associated with PCa bone metastasis, we performed high-density microarray analysis using fresh frozen PCa bone metastasis samples and here we present the genome-wide copy number change profiles for each sample to facilitate future collective research output. We found, in addition to other common genomic alterations previously reported in PCa, a common loss of 17q11.2, which was confirmed by fluorescence in situ hybridization (FISH) in a larger number of PCa bone metastasis samples. Our data further showed that the tumor suppressor microRNA located in this chromosomal region, miR-193a-3p, was commonly downregulated in PCa, leading to overexpression of cyclin D1 (*CCND1* gene) and uPA (*PLAU* gene) to promote cancer cell migration and invasion.

## 2. Materials and Methods

### 2.1. Patient Cohort

The patient sample cohort used in this study was described in Stankiewicz et al. [13]. Briefly, all samples were anonymized and obtained from Orchid Research Tissue Bank (HTA license number: 12199). Six fresh frozen and forty-three formalin-fixed paraffin-embedded (FFPE) PCa bone metastases were collected from The Robert Jones and Agnes Hunt Orthopaedic Hospital in Oswestry, Shropshire, from patients with advanced PCa who were treated for pathological bone fractures. Tissue-microarray was performed on 152 PCa and 55 benign prostatic hyperplasia (BPH) FFPE samples that were collected from patients at Barts Health NHS Trust in London. The study was performed under ethical approval from the East London and the City Research Ethics Committee. All tissue samples were reviewed by a consultant pathologist (Professor Daniel Berney).

### 2.2. Cell Lines

Human prostate cancer cell lines, PC3 (from a bone metastasis), 22RV1 (from primary PCa), PC3M-luc (a luciferase-expressing cell line that was derived from PC3M cells, a metastasis-derived variant of PC3), DU145 (from a brain metastasis), LNCaP (from a lymph node metastasis), and VCaP (from a vertebral metastasis) were used in this study. PC3, 22RV1, DU145, and VCaP were maintained in Dulbecco’s MEM (DMEM) (Sigma Aldrich, Gillingham, UK) containing 10% fetal bovine serum (FBS) and 1% penicillin/streptomycin (100 units/mL, Sigma Aldrich). LNCaP was maintained in RPMI-1640 Medium (Sigma Aldrich) containing 10% FBS and 1% penicillin/streptomycin (100 units/mL, Sigma Aldrich). The PC3M-luc cells were grown in MEM/BSS (Hyclone, Cytiva, India) supplemented with 10% FBS, non-essential amino acids (Hyclone), L-Glutamine (Hyclone), Na Pynrvate (Hyclone), and MEM Vitamin solution (Life Technologies, Paisley, UK). All cell lines were verified by microsatellite short tandem repeat (STR) profiling using the ABI AmpF/STR Identifiler kit (Life technologies).

### 2.3. SNP Array Analysis

DNA was extracted from six fresh frozen bone metastases. Samples preparation for hybridization onto Affymetrix SNP 6.0 array chips (Affymetrix, Santa Clara, CA, USA) was performed according to the manufacturer’s protocols. Signal intensity data from SNP arrays were analyzed using allele-specific copy number analysis of tumors (ASCAT v2.1) software [24]. ASCAT allows allele-specific copy number assessment, is less sensitive to noise in the input data, and corrects for both tumor cell aneuploidy and non-aberrant cell admixture, and therefore shows higher sensitivity to focal deletions.

### 2.4. Fluorescence In Situ Hybridization (FISH) Analysis

FISH was performed as previously described [13]. FISH probes were generated using the following Bacterial Artificial Chromosomes (BAC): RP11-398A1, CTD-2601E5, CTD-2283L18, CTD-2517E10, and RP11-958L7 for 17q11 lost region (labeled in red), and paracentromere of chromosome 1 was used as a control (labeled in green). Both FISH probe signals were counted per nucleus. A minimum of 100 nuclei (cells) with clear hybridization signals were counted per tissue sample and the percentage of nuclei with less 17q11 signals than control signals was determined. Cutoff for 17q11 loss was calculated as the mean of false-positive findings in ten non-malignant controls (BPH) plus three times the standard deviation (mean % ± 3SD) [25,26].

### 2.5. Quantitative Real-Time PCR (qRT-PCR)

Total RNA was extracted with TRIzol reagent (Life Technologies) according to manufacturer’s instructions. 30 ng was used for miR-193a-3p and RNU6B (endogenous control) cDNA synthesis with TaqMan MicroRNA Assays and MicroRNA Reverse Transcription Kit (Applied Biosystems, Carlsbad, CA, USA) according to supplied protocol. For regular cDNA synthesis, 1 µg of total RNA per sample was used with Moloney Murine Leukemia Virus Reverse Transcriptase, RNase H Minus, Point Mutant kit (Promega, Southampton, UK). All TaqMan assays were performed according to the manufacturer’s protocols (Life technologies). Standard qRT-PCR was performed using predesigned TaqMan gene expression assays: *PLAU*, *CCND1*, and *GAPDH* (endogenous control) (Life technologies).

### 2.6. Overexpression of miR-193a-3p

For transient miR-193a-3p overexpression, 130,000 (PC3) or 190,000 (22RV1) cells/well were seeded in 6-well plate and transfected with 25 nmol of mirVana miR-193a-3p mimic or miRNA mimic and 7.5 µL of Lipofectamine RNAiMAX Reagent in plain medium. RNA was extracted for qRT-PCR miR-193a-3p level analysis 24 h after transfection. For constitutive miR-193a-3p overexpression, 100,000 cells/well in 96-well plate were transduced with 16 × 10^6^ particles of shMIMIC human lentiviral microRNA hsa-miR-193a-3p with hCMV promoter and turbo GFP, or SMARTvector Non-targeting Control 1 in serum-free medium containing 14 µL/mL of polybrene. After transfection, cells were cultured in 2 µg/mL puromycin-containing medium for stable clone selection.

### 2.7. Gene Knockdown by Small Interfering RNA (siRNA)

60,000 PC3 cells/well were seeded in 6-well plates and transfected with 100 nM of *PLAU* and *CCND1*-specific smart pool siRNAs and control non-targeting siRNA (GE Dharmacon, Layfayette, CO, USA) using oligofectamine reagent (Life technologies) according to the manufacturer’s protocols.

### 2.8. Western Blotting

Western blotting was performed as previously described [13] using anti-urokinase (ab24121, Abcam, Cambridge, UK, 1:1000), anti-cyclin D1 (DCS-6, sc-20044, Santa Cruz, CA, USA, 1:500), anti-Mcl-1 (D5V5L, Cell Signaling, Danvers, MA, USA, 39224, 1:1000), anti-RAB27B (ab103418, Abcam), anti-PI3 Kinase p110α (C73F8, Cell Signaling 4249, 1:1000), anti-phospho-PI3 Kinase p85—Tyr458/p55—Tyr199 (Cell Signaling, 4228, 1:1000), anti-E-cadherin (Clone HECD-1, Takara, San Jose, CA, USA, M106, 1:1000), anti-N-cadherin (5D5, Abcam, ab98952, 1:1000), anti-vimentin (EP21, Epitomics, Burlingame, CA, USA, 1:3000), anti-CK18 (DC10, NCL-CK18, Novocastra, Newcastle Upon Tyne, UK, 1:3000) and anti-β-Actin (clone AC-15, A5441, Sigma Aldrich, 1:10,000) antibody.

### 2.9. Cell Viability, Migration, and Invasion Assays

Cell viability and proliferation were determined using the CellTiter 96 AQueous Cell Proliferation Assay (MTS) (Promega). In total, 5000 (PC3) or 10,000 (22RV1) cells/well were seeded in 96-well plates and assay was performed according to the manufacturer’s instructions. In vitro transwell migration and Matrigel invasion assays were carried out using 24-well, 8 µm pore transwell inserts (Becton Dickinson, Wokingham, UK). Cells, at 48 h post transfection with mimics/siRNA, were detached and re-seeded in plain medium into plain/Matrigel covered inserts at 50,000 (PC3) and 80,000 (22RV1) cells/insert, and 10% serum containing medium or MG63 cells (for PC3) was used as chemoattractant. Cells were allowed to migrate/invade for 24 h. Three inserts were used per condition.

### 2.10. Immunohistochemistry of Patient PCa Tissue Samples

FFPE clinical samples were cut at 4 µm with microtome (Leica, London, UK) and dewaxed with 2 changes in xylene (5 min each) and rehydrated through a series of alcohols. Endogenous peroxidise was blocked by incubation in 3% H_2_O_2_ solution in methanol for 10 min. Antigen retrieval was performed by microwaving the slides in TRIS-based, high pH solution (Vectastain, Vector Laboratories, Peterborough, UK) for 15 min. Slides were then incubated at room temperature for 1 h with cyclin D1 primary antibody (rabbit monoclonal, clone SP4, Vector Laboratories, Manchester, UK) diluted 1:50 in PBS with 1% BSA and secondary biotinylated universal antibody (Vectastain Universal Elite ABC Kit, Vector Laboratories) diluted in PBS. Finally, slides were incubated with avidin-peroxidise complex (Vectastain Universal Elite ABC Kit, Vector Laboratories) for 20 min and color development was performed by applying 3,3′-Diaminobenzidine (DAB) solution (BioGenex, Silsoe, UK), before counterstaining with hematoxylin. Cyclin D1 nuclear expression was scored by pathologist Daniel Berney for staining intensity (score 1—weak, 2—medium, 3—strong).

### 2.11. Statistical Analysis

Statistical tests included the chi-squared (*X*^2^) for FISH data comparison, the Mann–Whitney U test for FISH and cyclin D1 comparison and paired Student’s *t*-test for functional studies. The assumption of normality for *t*-tests was confirmed with Shapiro–Wilk test. Statistical analyses were performed using the Prism 5.0b (GraphPad, La Jolla, CA, USA) software. Values of *p* < 0.05 were considered significant.

## 3. Results

### 3.1. Loss of 17q11.2 Is Common in PCa Bone Metastases

**Figure 1:**
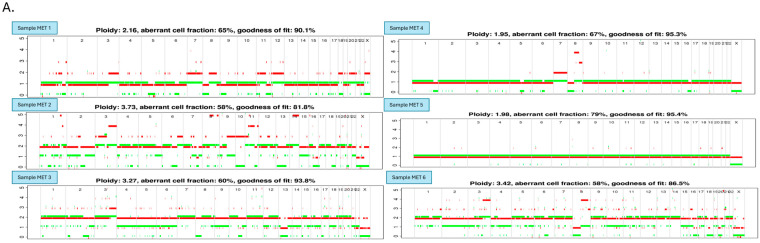

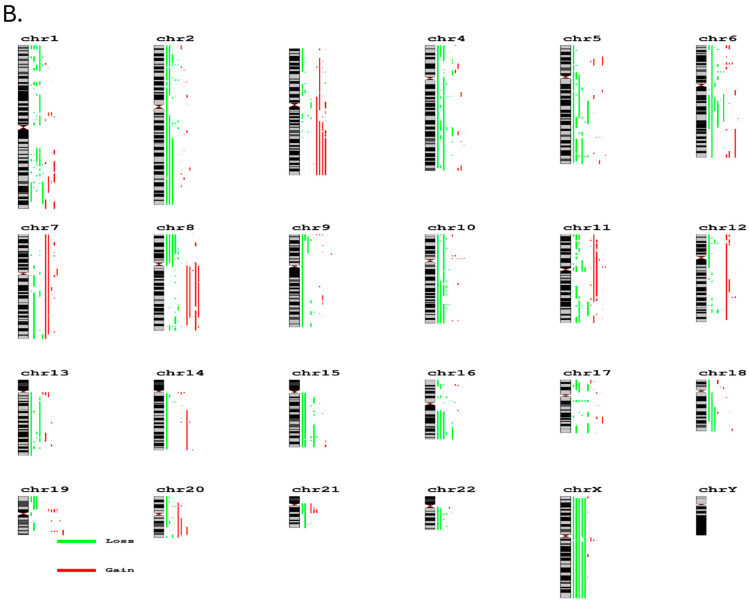
The genomic copy number alteration profiles of the six bone metastasis samples. (**A**) The allele-specific copy number changes along the human chromosomes for each of the six fresh frozen bone metastasis samples generated by ASCAT analysis. (**B**) The ideogram summarizes the gained and lost chromosomal regions in the six cases (ordered from left to right for cases 1 to 6) of bone metastasis samples along the human chromosomes. Losses are depicted in green and gains in red. MET: bone metastasis; chr: chromosome.

We performed allele-specific copy number analysis using the previously generated Affymetrix SNP arrays 6.0 data of six fresh frozen PCa bone metastasis samples (Gene Expression Omnibus accession no. GSE317433) coming from independent patients using the ASCAT software v2.1, which is a reliable and commonly used allele-specific copy number analysis software [24,27,28]. We identified many chromosomal region gains or losses which occurred in three or more out of the six cases, including gains on 1q32.2, 3p21.3, 3p22, 3q, 5p14, 5q31.3, 6p22.1-22.2, 6q27, 7, 8q, 9p24.3, 10p11.2, 11, 12q23.1, 13q12.1, 14q11.2, 19p11013.1, 19q13.4, 20q, 21q21.1-21.2, and loss of many regions or whole arms of 1p, 1q, 2p, 2q, 4p, 4q, 5q, 6q, 8p, 10q, 13q, 15q, 16q, 17p, 18q, 19p, 22q, and small regions of 3p3p13, 6p25, 6p21.1, 7q21.11, 8q24.3, 9p23-24.2, 9p21.2, 9q33.1, 10p12.3, 10p14-15, 11p15.5, 11p11-q12.1, 11q22.3-23.2, 12p13.1, 12q24.21, 14q13.1, 16p13.3, 16p12.1, 17q11.2, 20p13, 20p12.1, and 21q21.1 (Figure 1).

**Figure 2:**
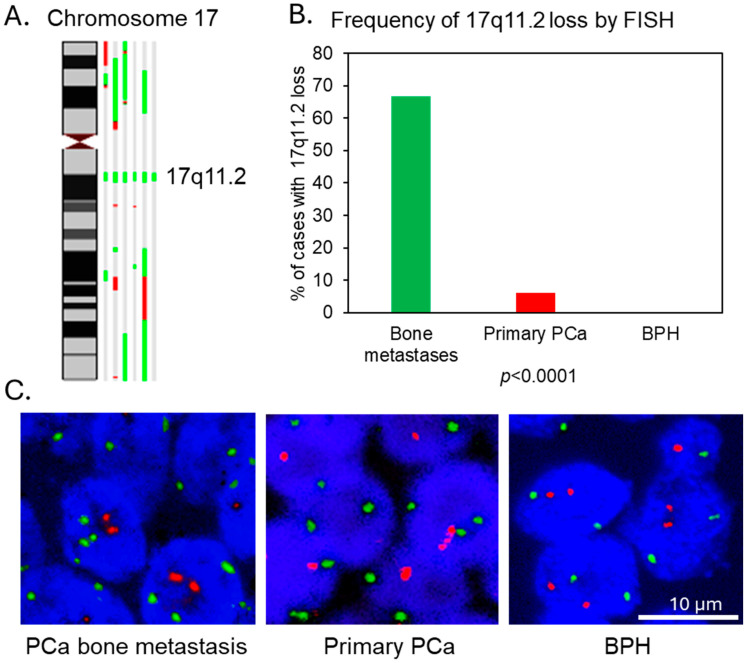
17q11.2 copy number loss in PCa samples. (**A**) Genomic copy number changes on chromosome 17 with marked 17q11.2 loss in six PCa bone metastases analyzed by SNP array. Losses are depicted in green and gains in red. (**B**) FISH analysis shows 17q11.2 copy number loss in the majority of bone metastases and a small subset of primary cases but not in non-malignant BPH controls. (**C**) Examples of 17q11.2 copy number loss in PCa, and normal 17q11.2 copy number status in BPH sample. Red, 17q11.2 region probe; green, a control probe; blue, nuclei. Nuclei with less red signals than green are considered as positive for 17q11.2 copy number loss.

Importantly, using the ASCAT software, we identified a small consistent chromosomal region loss on 17q11.2 in all the six PCa bone metastasis cases (Figure 2A) with minimal overlapping region between 31,490,881 and 33,142,272 bp (1,651,392 bp in total), which was missing from our previous data analysis of the same samples using our in-house GOLF (v2.2.10) software [13].

To confirm the high frequency of 17q11.2 loss, we successfully performed FISH analysis on an additional 21 cases of FFPE bone metastases tissue sections and detected loss of 17q11.2 in two thirds (14/21) of the samples (Figure 2B). We further analyzed this genomic region using FISH in 151 localized PCa and 55 benign prostate hyperplasia cases and only found 17q11.2 loss in 9 localized PCa cases (9/151), which is much rarer than in bone metastasis samples (*p* < 0.0001), and none in BPH samples (Figure 2B,C). Our Affymetrix SNP arrays 6.0 data of five PCa cell lines also showed that this 17q11.2 region was lost in the bone metastasis-derived PC3 PCa cell line by a large chromosomal region loss which included the whole 17p to 17q21.31 region, but was not lost in the PCa cell lines generated from soft tissue metastases (DU145 and LNCaP) or from the primary tumor (22RV1). However, copy number gain of this region was present in the bone metastasis-derived VCaP cell line (Figure S1).

### 3.2. Downregulation of the Tumor-Suppressing miRNA miR-193a-3p Is a Potential Driver Associated with the 17q11.2 Loss in PCa Metastasis

According to the Ensembl genome browser, there were 16 protein coding genes, 4 pseudogenes, 5 miRNAs, and 15 LncRNAs located in this 1,651,392 bp 17q11.2 lost genomic region (Table S1). Among the 16 protein coding genes, 9 of them have been mainly reported as oncogenes and the remaining 7 genes have not been reported with functional roles in cancer development. None of these pseudogenes and LncRNAs have been reported for their involvement in cancer. Interestingly, four out of the five miRNAs have been reported in cancer studies. The miR-193a, particularly miR-193a-3p, has been well established for its tumor-suppressing role [29,30,31,32,33,34,35,36,37,38,39] and miR-362 has been supported by many studies as an oncogene in several types of cancers [40,41,42,43,44,45,46,47,48,49,50,51], although also reported for potential tumor-suppressing role in a few studies [40]. The miR-365b has been reported for its tumor-suppressing role in one study [52] and for its oncogenic role in two other studies [53,54]. No abnormal expression has been reported for miR-4725, although a study showed that miR-4725 reduced cancer cell invasion when its expression was induced by a plant-derived drug [55] (Table S1). Based on the above data, we looked at RNA expression of miR-193a-3p and miR-365b by qRT-PCR in fresh-frozen tissue samples, including five of the six bone metastasis cases analyzed by SNP array, for which good quality RNA was available, four cases of localized primary PCa, and five cases of non-cancer control BPH samples, as well as five PCa cell lines. We detected low level expression of miR-365b in both non-malignant prostate tissue and the bone metastasis samples, much lower than in the PCa cell lines. Therefore, it is unlikely a targeted driver of 17q11.2 loss (Figure S2A). In contrast, miR-193a-3p was highly expressed in non-malignant prostate tissue samples but greatly reduced in four of the five bone metastasis samples analyzed. Surprisingly, miR-193a-3p was also expressed at a very low level, even lower than in the bone metastasis samples, in localized PCa (Figure S2B), which may be explained by other miR-193a-3p downregulation mechanisms frequently reported in the literature [29,30]. The bone metastasis-derived PCa PC3 cell line with 17q11.2 loss expressed lower level of miR-193a-3p than the other three metastasis-derived PCa cell lines without 17q11.2 region loss, but not the primary PCa tumor-derived 22RV1 cell line with normal copy number of 17q11.2, consistent with the findings in clinical samples. The bone metastasis-derived VCaP cell line with 17q11.2 gain expressed less miR-193a-3p than the copy number neutral soft tissue metastasis-derived DU-145 and LNCaP cell lines, indicating possible epigenetic suppression of miR-193a-3p expression in VCaP cells.

**Figure 3:**
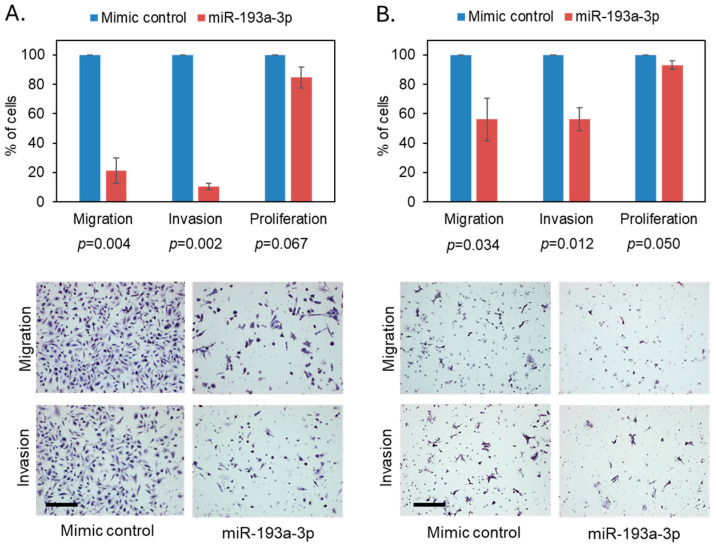
Overexpression of miR-193a-3p reduces PC3 and 22RV1 PCa migration and invasion. Bar chart and representative images show that miR-193a-3p overexpression in PC3 cells strongly inhibits cell migration and invasion (**A**), with a modest effect on cell viability. Bar chart and representative images show that miR-193a-3p overexpression in 22RV1 cells significantly inhibits cell migration and invasion but has no effect on proliferation (**B**). Scale bar: 100 μm.

To determine whether miR-193a-3p influences PCa metastasis, we overexpressed it using a mirVana™ miR-193a-3p mimic in 22RV1 (primary tumor origin, low metastatic potential, and normal copy of 17q11.2) and PC3 (bone metastasis origin with 17q11.2 loss) PCa cells, and confirmed overexpression by qRT-PCR (Figure S3). We found a significant reduction in PC3 cell migration and invasion (80% and 90% reduction, respectively), while it had minimal effect on their viability (Figure 3A). In 22RV1 cells, miR-193a-3p overexpression also significantly reduced cell migration and invasion, albeit not to the extent seen in PC3 cells (Figure 3B), where the baseline miR-193a-3p expression was higher than in 22RV1, indicating a potential link between genomic loss of the 17q11.2 region and cell sensitivity to miR-193a-3p inhibition effect.

### 3.3. The miR-193a-3p Suppresses PCa Cell Migration and Invasion Through Downregulation of Its Two Downstream Targets Cyclin D1 and uPA

**Figure 4:**
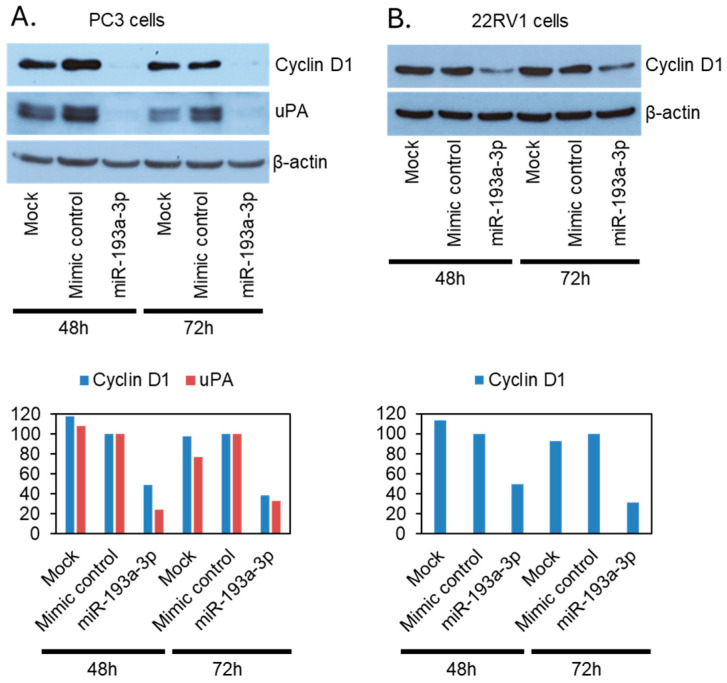
Overexpression of miR-193a-3p leads to downregulation of cyclin D1 and uPA in PCa cell lines. (**A**) Western blot analysis in PC3 cells reveals almost complete loss of cyclin D1 and uPA upon overexpression of miR-193a-3p. (**B**) 22RV1 cells exhibit a strong reduction in cyclin D1 protein expression upon overexpression of miR-193a-3p. 22RV1 cells do not express uPA. Mock, mock transfection; Mimic control, control cells transfected with non-targeting mimics; miR-193a-3p, cells transfected with miR-193a-3p mimics.

**Figure 5:**
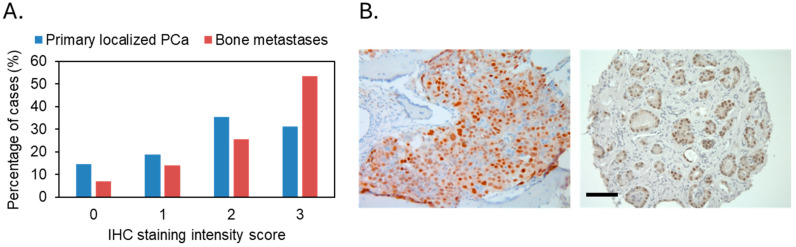
Cyclin D1 expression in the bone metastasis and localized PCa clinical samples. (**A**) Bar chart of the percentage of cases for each of the four immunohistochemistry staining intensity groups (0, 1, 2, and 3); (**B**) Representative immunohistochemistry staining images (left, bone metastasis; right, primary cancer). Mets: metastasis. Scale bar: 100 µm.

Many direct targets of miR-193a-3p have been reported in a cancer type-specific manner, including cyclin D1, uPA, Dnmt3a, Rab27b, HMGB1, and Mcl-1 [29,30]. To determine the target genes of miR-193a-3p in PCa, we analyzed the expression of the above six candidate gene proteins by Western blotting in the miR-193a-3p overexpressing PC3 and 22RV1 cells, comparing with the mock transfection and non-targeting miRNA control cells. Among the above-mentioned genes, cyclin D1 and uPA expression were strongly suppressed by miR-193a-3p overexpression in the PCa cells, except uPA in 22RV1 cells, where it was not expressed at all (Figure 4). None of the common epithelial–mesenchymal transition marker proteins were affected (Figure S4). We generated stable miR-193a-3p overexpressing PC3M (strongly metastatic clone of PC3) clones together with non-targeting control microRNA clones (Figure S5) and further confirmed the limited impact of miR-193a-3p overexpression on the protein expression of the other miR-193a-3p target genes, Dnmt3a, Rab27b, HMGB1, and Mcl-1 (Figure S6). We analyzed cyclin D1 expression in the bone metastasis and localized PCa clinical samples by immunohistochemistry. In the bone metastasis cohort, only 3 of the 43 cases were negative for cyclin D1 while 23/43 were strongly stained (level III intensity). In the localized cancer cohort, 22 out of the 152 cases were cyclin D1 negative while only 49/152 were strongly stained (level III intensity). Cyclin D1 was statistically (*p* = 0.013) less strongly expressed in localized PCa than bone metastasis (Figure 5) as well as in PCa cases without 17q11.2 loss when compared with cases positive for 17q11.2 loss (*p* = 0.035, Figure S7). While above results confirmed that cyclin D1 was highly expressed in both PCa bone metastasis and primary tumors, it seems that there are other miR-193a-3p genomic losses associated, in addition to miR-193a-3p expression level-related, with cyclin D1 expression inhibition during bone metastasis.

When we depleted *CCND1* and *PLAU* via siRNA-mediated knockdown in PC3 cells, as expected we also observed a significant inhibition of cell migration and invasion, especially upon uPA downregulation (Figure S8).

## 4. Discussion

There are limited studies on genome-wide profiles at an individual sample level for genomic copy number changes in bone metastases of PCa despite their high frequency. We produced high-resolution genomic copy number changes for six bone metastasis samples for the collective effort to identify bone metastasis specific genomic changes. We identified a <2 mb genomic loss on 17q11.2 in all the samples. The high frequency of this genomic loss, which contains a well-established tumor-suppressing miRNA, miR-193a-3p, was further confirmed in a larger cohort of bone metastasis FFPE samples by FISH. The cell invasion suppressing role as well as target genes of miR-193a-3p in PCa were investigated, identifying *CCND1* and *PLAU* as the main target genes.

Due to the difficulty in obtaining bone metastasis samples, bone metastasizing PCa studies are few in number despite their frequency and their impact on health, morbidity and mortality worldwide [9,10,11,12,13,14,15,16,17,18,19,20,21,22,23]. The limited studies available using bone metastasis samples rarely include individual sample levels for genomic copy number changes (the most common genomic alterations in PCa) [9,10,11,12,13,14,15,16]. Although the case number is limited, here we present the profiles of high-resolution genomic copy number changes for six bone metastasis samples. They show that bone metastases share the common genetic alterations with primary tumors and other non-bone metastases, such as gains of 3q, 7, 8q, losses of 5q, 6q, 8p, 10q, 13q, 16q, 17p, 18q, and 22 [10,11,17,19,20,21]. There are also potential common genetic alterations specific to PCa bone metastasis, including certain regional losses on 1q, 2, 4q, 17q, and 19p.

Most importantly, we identified a high frequency loss of a small chromosome region on 17q11.2, which was confirmed by FISH in a larger number of bone metastasis samples. With many oncogenes located on it, 17q is more commonly gained than lost in human malignancies, including PCa [11,56,57,58,59,60,61]. However, the loss covering 17q11.2 has been reported in some studies of colorectal and breast cancers and osteosarcoma, linking it to their metastatic abilities [62,63,64,65]. In the minimal overlapping lost genomic region defined by our SNP array data, there are several genes and non-coding RNAs previously reported as oncogenes or having oncogenic roles (Table S1). Only miR-193a-3p is a well-established tumor suppressor reported in many cancer types, including prostate, breast, colon, bladder, and lung cancers, and melanoma and hematological malignancies [29,30,33,34,35,36,37,38,39]. Therefore, based on the existing literature and our gene expression analysis, miR-193a-3p is a potential key target of 17q11.2 loss.

MicroRNAs are endogenous 18-24-nucleotide non-coding RNA molecules that regulate the expression of many protein-coding genes through inhibition of translation initiation and degradation of their target mRNAs. Dysregulation of miRNA expression is a common hallmark in cancers and leads to abnormal expression of many genes [66]. Many direct targets of miR-193a-3p have been reported in a cancer type-specific manner [29,30]. The miR-193a-3p suppresses the metastasis of osteosarcoma cells by down-regulating Rab27B [54], which is consistent with the loss of 17q reported in osteosarcoma metastasis [63]. In breast cancer, miR-193a-3p was shown to inhibit cancer cell migration and invasion through direct degradation of its target genes *CCND1* (coding for cyclin D1) and *PLAU* (coding for uPA) [67], which are commonly overexpressed in metastatic PCa [68]. In keeping with our results, *CCND1* has also been confirmed as miR-193a-3p target gene in PCa and stable miR-193a-3p transfection resulted in inhibition of cell viability, proliferation, and colony formation, and induced G1 phase arrest in PC3 and DU145 metastatic PCa cells [32]. Additionally, we showed, by immunohistochemical staining, that the protein coded by *CCND1*, cyclin D1, was highly expressed in PCa bone metastasis samples.

The uPA, together with its receptor uPAR, and plasminogen activator inhibitor-1 and -2 (PAI-1 and PAI-2) forms the plasminogen activator (PA) system, an extracellular proteolytic enzyme system involved in normal physiological processes like fibrin clot dissolution, wound healing, and inflammatory responses. uPA converts inactive plasminogen into plasmin, which degrades surrounding tissue. In malignancies, uPA and uPAR are often overexpressed and involved in proteolytic digestion of the extracellular matrix, resulting in tumor cell migration, invasion, and metastasis [69]. The expression of uPAR is often localized in a leading edge of migrating cells allowing high local concentration of uPA and therefore focused plasmin-mediated extracellular matrix degradation facilitating cell migration [70,71]. Consistent with our data, it has been reported that blocking of cell surface uPA or uPAR activity or knockdown of uPA and uPAR in PC3 cells significantly inhibited their invasion, survival, tumorigenicity, and metastatic capacity in vitro and in vivo [72,73]. PCa cell-derived uPA has been reported to contribute to intraosseous tumor growth and bone turnover [74]. Tumor-derived uPA has also been shown to be involved in immunosuppression by recruiting myeloid-derived suppressor cells (MDSCs) capable of suppressing T cells, and different levels of tumor-derived uPA correlated with MDSC recruitment to tumor cells and tumor development [75]. In PCa patients, significant increase in uPA and uPAR levels has been observed from patients with non-metastatic PCa to patients with lymph node metastases and patients with skeletal metastases [76]. Additionally, high levels of uPA and uPAR in the plasma significantly correlated with increased aggressiveness, postoperative progression, and metastasis. Patients with high-risk and/or metastatic PCa have been reported to have significantly higher expression levels of uPAR in disseminated tumor cells (DTC) in their bone marrow than patients with low-risk disease and the presence of uPAR-positive DTC in bone marrow correlated with cancer relapse [77]. Therefore, the role of cyclin D1 and uPA in PCa progression, including bone metastasis, has already been supported by many studies. This study linked them to our novel finding of the high frequency loss of 17q11.2 genomic region through the downregulation of miR-193a-3p expression and ruled out some other reported miR-193a-3p target genes for their involvement in PCa when miR-193a-3p expression was downregulated.

Although in PCa bone metastasis, the 17q11.2 loss may be accountable for the reduced miR-193a-3p expression, paradoxically, miR-193a-3p expression was also downregulated in localized PCa samples. In fact, miR-193a-3p expression is regulated by DNA methylation and epigenetic change-induced miR-193a-3p downregulation has been frequently reported, and is more common than genomic loss-induced downregulation [29,30]. This could explain why primary and bone metastasis samples have similar miR-193a-3p expression in our sample cohorts. However, the genomic loss of miR-193a-3p seems to affect its target gene regulation in addition to miR-193a-3p expression level. The protein expression of the well-established miR-193a-3p target gene in PCa, *CCND1*, was more deregulated in the bone metastasis than primary PCa samples, which had similarly low level of miR-193a-3p expression. This is consistent with our observed miR-193a-3p cellular function in suppressing PCa cell migration and invasion, where overexpression of miR-193a-3p suppressed PC3 cell (positive for 17q11.2 loss) migration and invasion much more than 22RV1 cells (lack of 17q11.2 loss, though lower miR-193a-3p expression). The reasons why the genomic losses are required in addition to non-genomic mechanism of downregulation of miR-193a-3p require further investigation.

One of the potential reasons for the 17q11.2 genomic loss may be the natural selection of fitness of PCa cell clones during the interaction of PCa and osteoblast cells to facilitate bone metastasis development through the miR-193a-3p regulated uPA expression. The expression of both miR-193a-3p and uPA can be controlled by DNA methylation [29,30,78]. Downregulation of miR-193a-3p and uPA may have a high chance of both being induced by DNA methylation as occurred in the 22RV1 cell line, which was derived from a primary PCa with high aggressiveness in animal model [79] but with limited migration ability. It has been reported that osteoblast cells can induce PCa cell migration and invasion through enhancing uPA expression both by cell-to-cell direct contact and through osteoblast secreted factors [80,81]. In turn, overexpression of uPA in PCa cells can induce osteoblastic activity in the bone and enhance the ability of forming bone metastasis [82]. PCa cells without uPA expression, potentially caused by DNA methylation, may lack the ability to facilitate PCa and osteoblast interaction to form bone metastasis.

**Figure 6:**
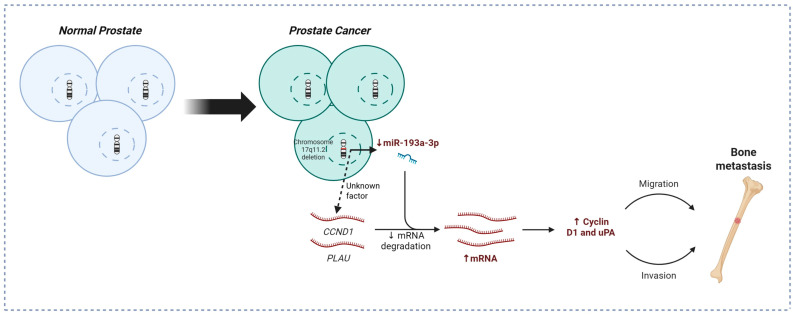
A brief schematic model summary of the proposed mechanism linking 17q11.2 loss, miR-193a-3p, CCND1/uPA, and bone metastasis. Created in BioRender. https://BioRender.com/r83g977 (accessed 14 January 2026).

Based on our study, we have proposed a model for the role of 17q11.2 loss in PCa bone metastasis (Figure 6), linking 17q11.2 loss to miR-193a-3p downregulation, subsequent *CCND1*/*PLAU* overexpression, and increased migration and invasion capabilities of affected cells. Of note, there are several limitations of this study. Firstly, due to the lack of and necessity for routine bone biopsies performed for clinical reasons, all the bone metastasis samples came from patients surgically treated for pathological bone fractures. It remains to be assessed if 17q11.2 loss is also frequently lost in early bone metastasis lesions without bone fracture. Secondly, although we have a substantial number of bone metastasis cases, considering their restricted availability, the number of cases is still limited, particularly for fresh frozen samples. Due to limited material from the six fresh frozen bone metastasis specimens we were unable to assess protein levels, including cyclin D1 and uPA in those samples. Furthermore, our study indicates that 17q11.2 loss may have additional impact on cyclin D1 expression and PCa bone metastasis to downregulation of miR-193a-3p, but the exact mechanism has yet to be investigated. Finally, while cyclin D1 and uPA are well-established target genes of miR-193a-3p, we only verified it in our study by in vitro overexpression of miR-193a-3p in PCa cell lines. Our proposed model requires further functional mechanistic investigation, such as targeted chromosome 17 loss, additional loss-of-function or rescue-based experiments, particularly using in vivo animal models, to validate the proposed bone metastasis model.

## 5. Conclusions

In summary, we have provided high-resolution bone metastasis profiles of individual bone metastasis cases to facilitate future research in this critical area of PCa. Through high-density SNP array common genomic alteration discovery and FISH confirmation in a larger number of bone metastasis samples, we identified a novel high frequency small genomic region loss on 17q11.2, which contains a well-established tumor-suppressing miRNA miR-193a-3p. Moreover, we showed that miR-193a-3p suppressed PCa cell migration and invasion by regulating cyclin D1 and uPA expression. Therefore, we provide new insight into bone metastasis development, which will hopefully facilitate the development of preventive and therapeutic approaches to control PCa bone metastasis, consequently improving PCa patients’ quality of life and clinical outcome.

## Data Availability

The original data presented in the study are openly available in Gene Expression Omnibus (accession no. GSE317433). Further inquiries can be directed to the corresponding author.

## Supplementary Materials

The following supporting information can be downloaded at: https://www.mdpi.com/article/10.3390/cancers18030403/s1, Figure S1: Chromosome 17 Affymetrix SNP arrays 6.0 data of the five PCa cell lines. Figure S2: MicroRNA expression in clinical samples and cell lines. Figure S3: Overexpression of miR-193a-3p in PCa cell lines. Figure S4: Overexpression of miR-193a-3p had no effect on common epithelial–mesenchymal transition marker proteins. Figure S5: The expression level of miR-193a-3p detected by qRT-PCR in stable miR-193a-3p overexpression PC3M clones together with non-targeting control microRNAs. Figure S6: Overexpression of miR-193a-3p has limited impact on Mcl-1 expression and no impact on HMGB1, Dnmt3a, or Rab27b expression in PC3M-luc PCa cells. Figure S7: Bar chart of the percentage of cases for each of the four immunohistochemistry staining intensity groups (0, 1, 2 and 3) in 17q11.2 loss and non-loss cases. Figure S8: Downregulation of cyclin D1 and uPA leads to reduced PCa cell migration and invasion. Table S1: Genes and Non-coding RNAs located in the lost 17q11.2 genomic regions and their reported roles in cancer.

## Abbreviations

The following abbreviations are used in this manuscript:

ASCAT	allele-specific copy number analysis of tumors
BAC	bacterial artificial chromosomes
BPH	benign prostatic hyperplasia
DMEM	Dulbecco’s Modified Eagle’s Medium
Dnmt3a	DNA Methyltransferase 3 Alpha
DTC	disseminated tumor cell
FBS	fetal bovine serum
FFPE	formalin-fixed paraffin-embedded
FISH	fluorescence in situ hybridization
HMGB1	High Mobility Group Box 1
LncRNA	long non-coding RNA
Mcl-1	Induced Myeloid Leukemia Cell Differentiation Protein
MDSC	myeloid-derived suppressor cell
PA	plasminogen activator
PCa	prostate cancer
PLAU	plasminogen activator, urokinase
qRT-PCR	quantitative Real Time PCR
Rab27b	Ras-Related Protein Rab-27B
SD	standard deviation
siRNA	small interfering RNA
STR	short tandem repeat
uPA	urokinase-type plasminogen activator
