# Supplementary material for: High Frequency Loss of 17q11.2 and Downregulation of the Cancer Metastasis Suppression microRNA miR-193a-3p in Prostate Cancer Bone Metastasis
Source: Cancers (Basel). 2026 Jan 27;18(3):403. doi: 10.3390/cancers18030403 (PMC12896823; doi:10.3390/cancers18030403)
Supplement: Supplementary file 1 [file cancers-18-00403-s001.zip › cancers-4107606-supplementary.pdf]

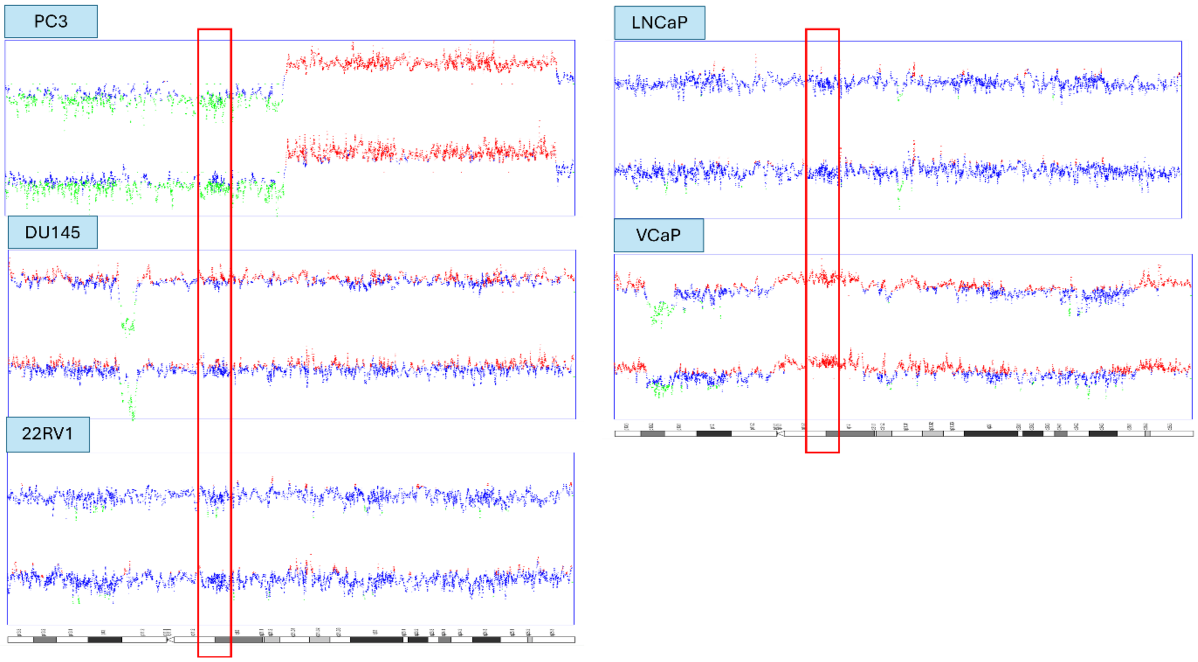

Supplementary Figure S1. Chromosome 17 Affymetrix SNP arrays 6.0 data of the five PCa cell lines. The 17q11.2 region was lost in PC3, gained in VCaP and did not change copy number in DU145, LNCaP and 22RV1 and VCaP.

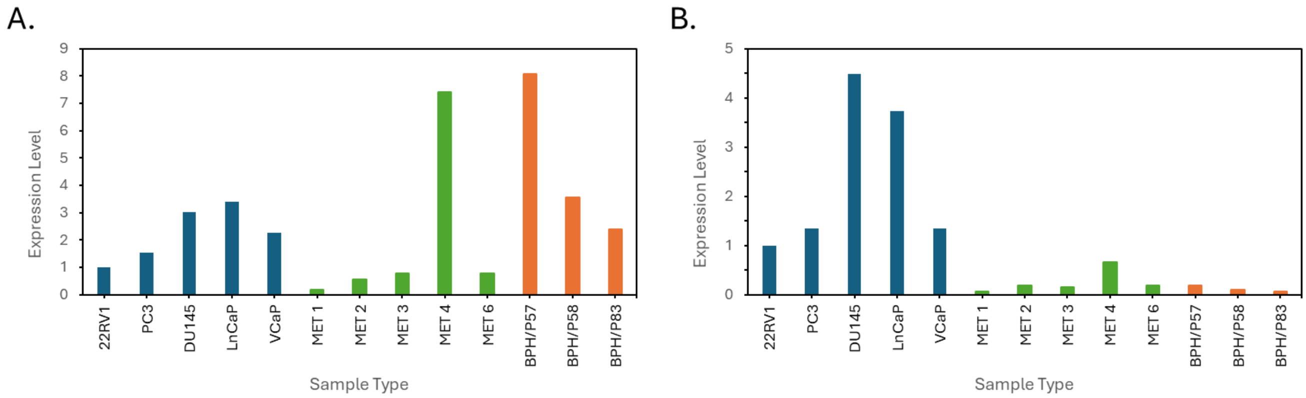

Supplementary Figure S2. MicroRNA expression in clinical samples and cell lines. The expression of (**A**) miR-193a-3p and (**B**) miR-365b detected by qRT-PCR in bone metastasis, primary PCa and BPH samples and PCa cell lines.

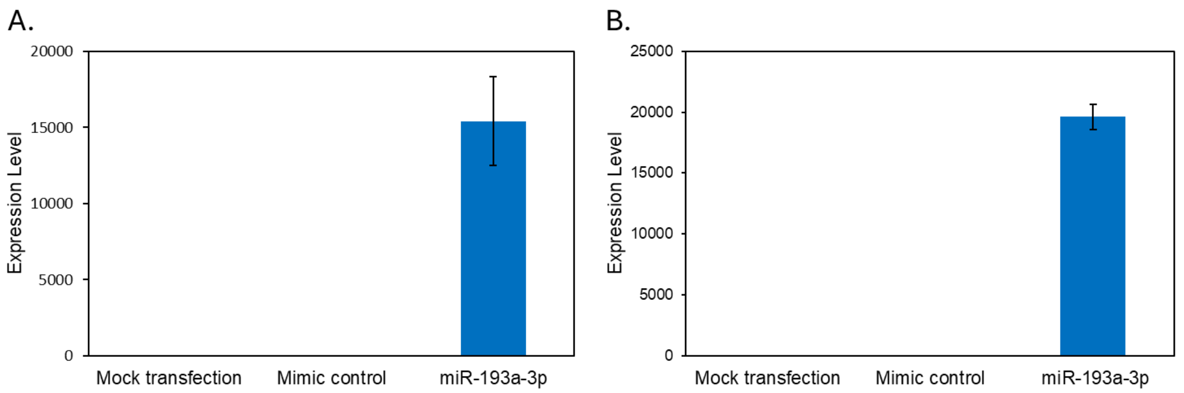

Supplementary Figure S3. Overexpression of miR-193a-3p in PCa cell lines. The expression of miR-193a-3p detected by qRT-PCR in PCa cell line (**A**) PC3 and (**B**) 22RV1 transfected with the miR-193a-3p overexpression construct and controls.

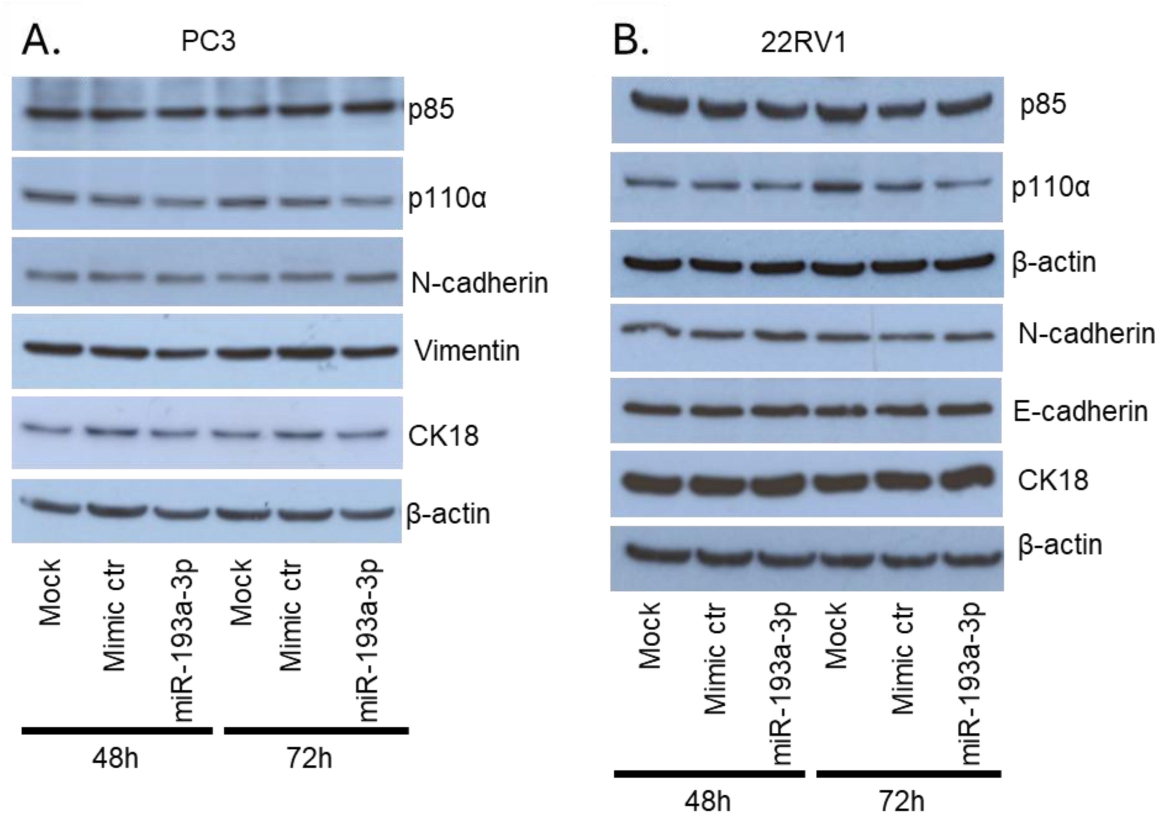

Supplementary Figure S4. Overexpression of miR-193a-3p had no effect on common epithelial-mesenchymal transition marker proteins. The expressions of epithelial-mesenchymal transition protein markers were detected by Western Blotting in PCa cell line (**A**) PC3 and (**B**) 22RV1 transfected with miR-193a-3p overexpression construct and controls.

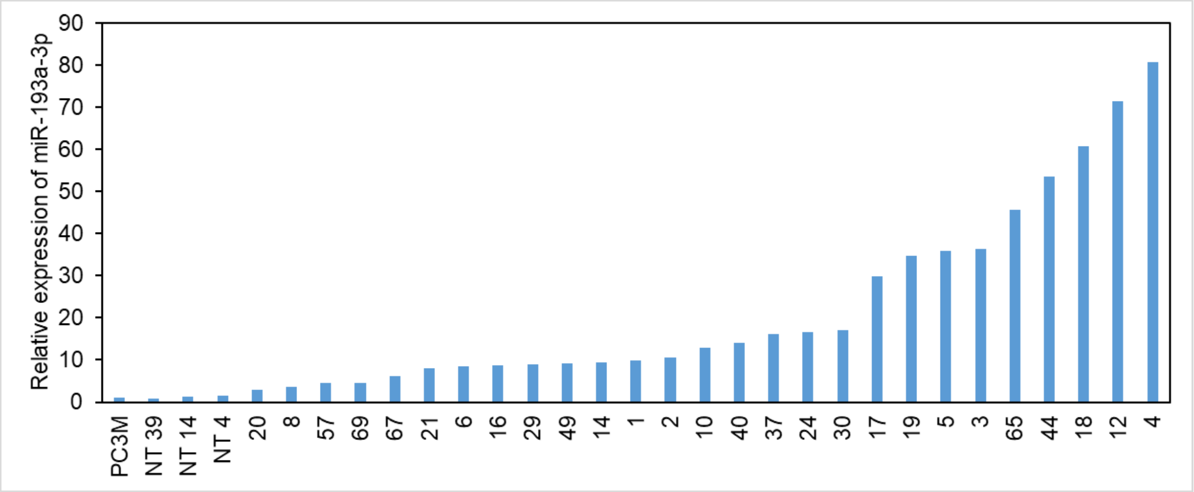

Supplementary Figure S5. The expression level of miR-193a-3p detected by qRT-PCR in stable miR-193a-3p overexpression PC3M clones together with non-targeting control microRNAs.

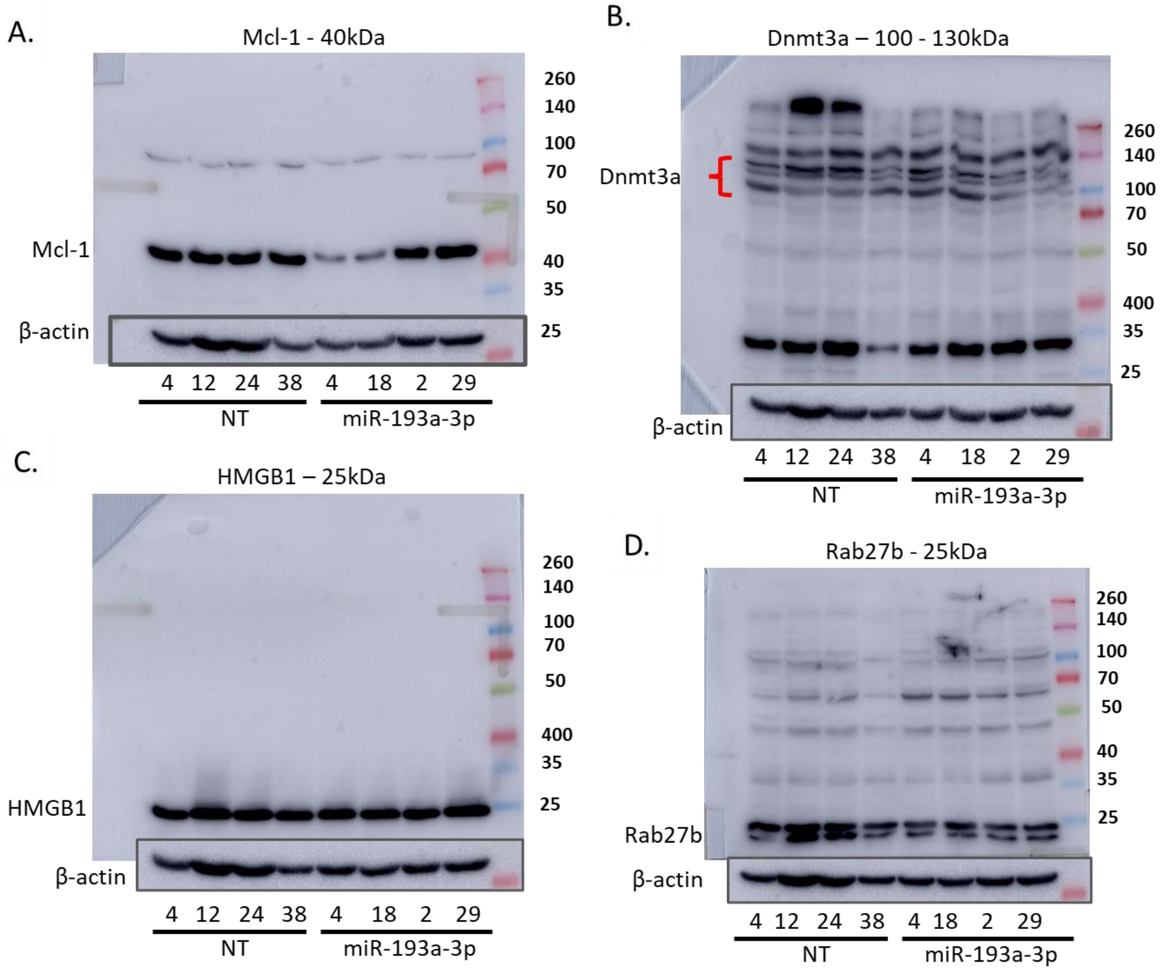

Supplementary Figure S6. Overexpression of miR-193a-3p has limited impact on (**A**) Mcl-1 expression and no impact on (**B**) Dnmt3a (**C**) HMGB1 or (**D**) Rab27b expression in PC3M-luc PCa cells. Western blot protein expression was performed on PC3M-luc single clones. NT, control cells transfected with non-targeting miRNA lentiviral vector; miR-193a-3p, cells transfected with miR-193a-3p expression lentiviral vector.

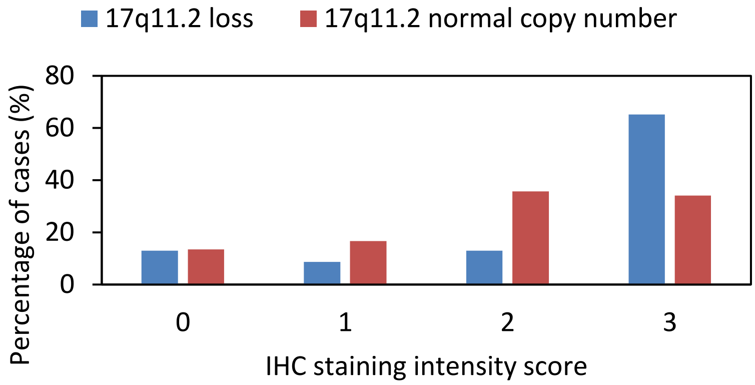

Supplementary Figure S7. Bar chart of the percentage of cases for each of the four immunohistochemistry staining intensity groups (0, 1, 2 and 3) in 17q11.2 loss and non-loss cases. Patients with 17q11.2 loss had a higher expression of cyclin D1 compared to patients with a normal copy number (p = 0.035).

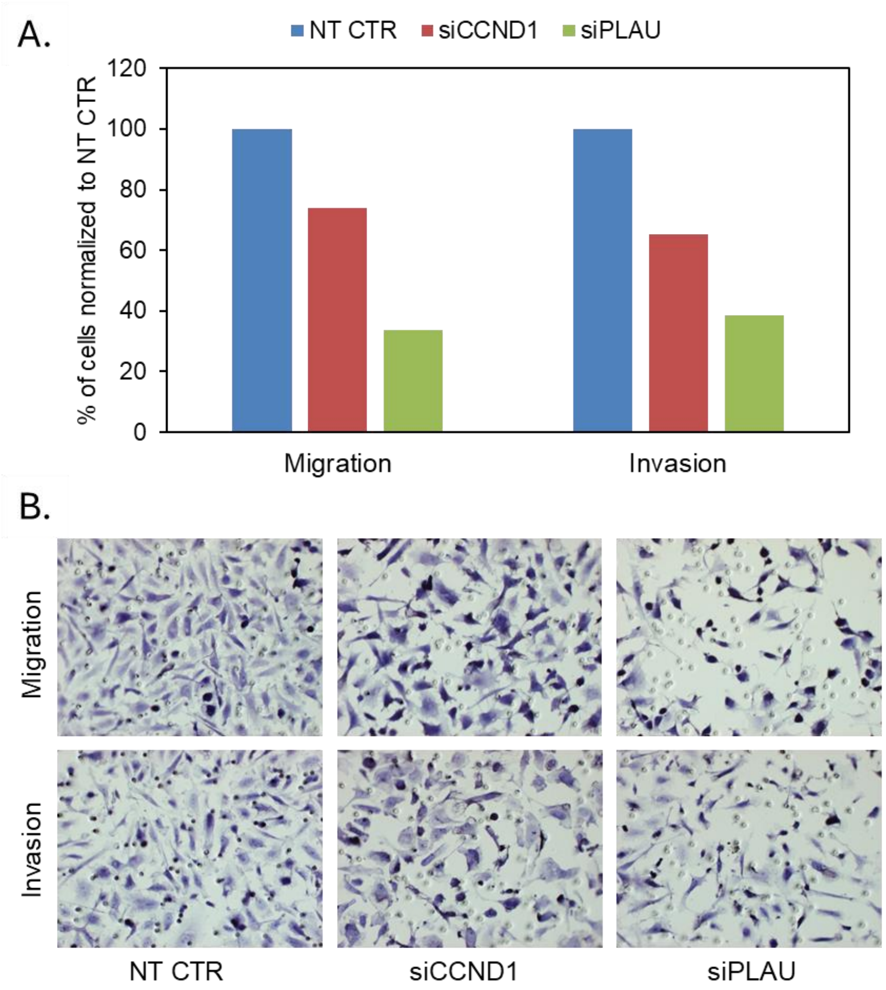

Supplementary Figure S8. Downregulation of cyclin D1 and uPA leads to reduced PCa cell migration and invasion. Knockdown of CCND1 or PLAU by siRNA resulted in significant reduction in PC3 transwell cell migration and matrigel invasion, with greater impact observed upon PLAU downregulation. (**A**) bar chart and (**B**) representative images.

Supplementary Table S1. Genes and Non-coding RNAs located in the lost 17q11.2 genomic regions and their reported roles in cancer.

Gene Name	Gene type	Ensembl Gene ID	Start (bp)	End (bp)	Strand	Gene Description	Oncogenic / Tumor Suppressor Evidence (DOI)
RAB11FIP4	protein coding	ENSG00000131242	31,391,675	31,538,211	+	RAB11 family interacting protein 4	Oncogenic evidence in serous ovarian cancer (10.1073/pnas.1508057112), oncogenic evidence in hepatocellular carcinoma (10.1007/s13105-023-00972-2 AND 10.1038/onc.2015.49), oncogenic evidence in pancreatic cancer (10.3892/ijo.2016.3828), oncogenic evidence in colorectal cancer (10.3892/mmr.2017.8283).
RN7SL45P	misc RNA	ENSG00000264862	31,518,432	31,518,729	+	RNA, 7SL, cytoplasmic 45, pseudogene	No data
TEC	TEC	ENSG00000278977	31,533,171	31,533,938	−	To be experimentally confirmed	No data
MIR4724	miRNA	ENSG00000284424	31,534,883	31,534,971	+	microRNA 4724	No data
lncRNA (novel)	lncRNA	ENSG00000300349	31,548,985	31,549,709	−	Novel long non-coding RNA	No data

MIR193A	miRNA	ENSG00000207614	31,559,996	31,560,083	+	microRNA 193a	Tumor suppressor evidence in PCa (10.3892/ol.2017.6865, 10.2147/CMAR.S190669), tumor suppressor evidence in breast cancer (10.1111/febs.14724), tumor suppressor evidence in colon cancer (10.1002/advs.202307937, 10.1158/1078-0432.CCR-17-0171), tumor suppressor evidence in lung cancer (10.1016/j.canlet.2019.01.027), tumor suppressor evidence in bladder cancer (10.1038/cddis.2014.367), tumor suppressor evidence in melanoma (10.3390/ijms21176183), tumor suppressor evidence in large B-cell lymphoma (10.1186/s12967-025-06689-8); two review papers (10.1155/2017/5913195, 10.1002/jcp.28368).
lncRNA (novel)	lncRNA	ENSG00000278546	31,560,132	31,560,573	+	Novel long non-coding RNA	No data
RNU6ATAC7P	snRNA	ENSG00000221038	31,563,768	31,563,894	−	RNA, U6atac small nuclear 7, pseudogene	No data
MIR365BHG	lncRNA	ENSG00000228768	31,571,074	31,575,659	+	MIR365B and MIR4725 host gene	No data
MIR4725	miRNA	ENSG00000265976	31,575,269	31,575,358	+	microRNA 4725	No abnormal expression has been reported, but when its expression was induced by a drug in glioma cancer cells, it showed a tumor suppressing role (10.1111/jnc.14459).

MIR365B	miRNA	ENSG00000283978	31,575,411	31,575,521	+	microRNA 365b	Tumor suppressor evidence in Non-Small Cell Lung Cancer (10.3892/etm.2020.8857), oncogenic evidence in hepatocellular carcinoma (10.2217/fon-2018-0676), oncogenic evidence in renal cell carcinoma (10.1186/s12964-024-02001-1).
lncRNA (novel)	lncRNA	ENSG00000266877	31,583,162	31,637,543	+	Novel long non-coding RNA	No data
lncRNA (novel)	lncRNA	ENSG00000309286	31,599,818	31,606,142	−	Novel long non-coding RNA	No data
lncRNA (novel)	lncRNA	ENSG00000309179	31,690,466	31,692,319	+	Novel long non-coding RNA	No data
lncRNA (novel)	lncRNA	ENSG00000297113	31,708,831	31,709,946	−	Novel long non-coding RNA	No data
MYT1L-P	processed pseudogene	ENSG00000266448	31,709,568	31,709,859	+	Myelin transcription factor 1-like pseudogene	No data
RNU6-1134P	snRNA	ENSG00000202026	31,713,753	31,713,853	+	RNA, U6 small nuclear 1134, pseudogene	No data
lncRNA (novel)	lncRNA	ENSG00000308319	31,725,345	31,727,073	+	Novel long non-coding RNA	No data

GPR160P2	processed pseudogene	ENSG00000265863	31,759,795	31,761,385	+	GPR160 pseudogene 2	No data
lncRNA (novel)	lncRNA	ENSG00000263567	31,762,440	31,769,048	+	Novel long non-coding RNA	No data
lncRNA (novel)	lncRNA	ENSG00000265046	31,830,731	31,831,750	+	Novel long non-coding RNA	No data
COPRS	protein coding	ENSG00000172301	31,851,871	31,859,291	−	Coordinator of PRMT5 and differentiation stimulator	No oncogenic/tumor suppressive evidence.
UTP6	protein coding	ENSG00000108651	31,860,904	31,901,746	−	UTP6 small subunit processome component	No oncogenic/tumor suppressive evidence.
lncRNA (novel)	lncRNA	ENSG00000263990	31,873,926	31,886,666	+	Novel long non-coding RNA	No data
SUZ12	protein coding	ENSG00000178691	31,936,995	32,001,045	+	Polycomb repressive complex 2 subunit	Oncogenic evidence in gastric cancer (10.1002/jbt.23365 AND 10.1007/s13277-015-3195-7 AND 10.18632/aging.203130), oncogenic evidence in head and neck squamous cell carcinoma (10.1111/jcmm.13638), tumor suppressor evidence in HBV-related hepatocellular carcinoma (10.7150/jca.29932).

RNA5SP437	rRNA pseudogene	ENSG00000253058	31,963,805	31,963,933	−	RNA, 5S ribosomal pseudogene 437	No data
BPTF-P	transcribed processed pseudogene	ENSG00000264300	32,003,110	32,003,326	−	Bromodomain PHD finger transcription factor pseudogene	No data
lncRNA (novel)	lncRNA	ENSG00000293286	32,003,203	32,006,688	−	Novel long non-coding RNA	No data
LRRC37B	protein coding	ENSG00000185158	32,007,383	32,053,504	+	Leucine rich repeat containing 37B	No data
lncRNA antisense LRRC37B	lncRNA	ENSG00000308099	32,046,427	32,067,365	−	Antisense to LRRC37B	No data
RHOT1/MIRO-1	protein coding	ENSG00000126858	32,142,442	32,253,374	+	Ras homolog family member T1	Tumor suppressor evidence through mitochondrial trafficking (10.1126/sciadv.adi4298), oncogenic evidence in breast cancer (10.1186/s12935-023-03111-5), oncogenic evidence in colorectal cancer (10.1038/s41598-023-40358-4), oncogenic evidence in pancreatic cancer (PMC4473323), oncogenic evidence in gastric cancer (10.3892/ijo.2025.5810).

RHBDL3	protein coding	ENSG00000141314	32,265,787	32,324,663	+	Rhomboid like 3	No oncogenic/tumor suppressive evidence.
C17orf75	protein coding	ENSG00000108666	32,324,565	32,350,023	−	Chromosome 17 open reading frame 75	No oncogenic/ tumor suppressive evidence.
MIR632	miRNA	ENSG00000283774	32,350,109	32,350,202	+	microRNA 632	Oncogenic evidence in breast cancer (10.3892/ol.2015.3993), oncogenic evidence in hepatocellular carcinoma (10.1089/humc.2019.040, 10.18632/aging.203939, 10.1016/j.canlet.2014.10.041), oncogenic evidence in gastric cancer (10.1186/1479-5876-12-33, 10.1186/s12885-018-5247-z), oncogenic evidence in bladder cancer (10.3389/fphar.2020.00164), oncogenic evidence in laryngeal cancer (10.3727/096504018X15213142076069), oncogenic evidence in glioblastoma (10.26355/eurrev_202009_22834), oncogenic evidence in myeloid leukemia (10.1007/s13577-019-00319-4, 10.1186/s12967-018-1445-3); a review paper (10.1007/s12032-025-02900-4).
ZNF207	protein coding	ENSG00000010244	32,350,122	32,381,885	+	Zinc finger protein 207	Tumor suppressor evidence as a regulator of kinetochore-microtubule attachment (10.1038/s44318-025-00469-2), oncogenic evidence in hepatocellular carcinoma (10.11817/j.issn.1672-7347.2019.04.010 AND 10.1016/j.cellsig.2025.112009),

PSMD11	protein coding	ENSG00000108671	32,444,379	32,483,319	+	Proteasome 26S subunit, non-ATPase 11	Oncogenic evidence in hepatocellular carcinoma (10.1016/j.cellsig.2024.111279 AND 10.1002/mc.23568), oncogenic evidence in lung adenocarcinoma (10.21037/jtd-24-1622).
CDK5R1	protein coding	ENSG00000176749	32,486,156	32,491,253	+	Cyclin-dependent kinase 5 regulatory subunit 1	Oncogenic evidence in colorectal and breast cancer (10.1016/j.cbi.2022.110190), oncogenic evidence in hepatocellular carcinoma (10.1042/BSR20203594), oncogenic evidence in lung adenocarcinoma (10.1038/s41598-025-87982-w).
MYO1D	protein coding	ENSG00000176658	32,492,522	32,877,192	−	Myosin ID	Oncogenic evidence in Triple-Negative Breast Cancer (10.1007/s00432-024-05651-3), oncogenic evidence in colorectal cancer (10.1038/s41388-019-0954-8), oncogenic evidence in Acute Myeloid Leukemia (10.1182/bloodadvances.2021006920).
MYO1D-AS1	lncRNA	ENSG00000264458	32,627,684	32,686,485	+	MYO1D antisense RNA 1	No oncogenic/tumor suppressive evidence.
H2BN1	protein coding	ENSG00000290320	32,895,433	32,906,586	+	H2B.N variant histone 1	No oncogenic/tumor suppressive evidence. Core component of nucleosome.
TMEM98	protein coding	ENSG00000006042	32,927,910	32,945,106	+	Transmembrane protein 98	Oncogenic evidence in lung, gastric, hepatic, ovarian, to head and neck cancer (10.1186/s12967-025-06998-y), oncogenic evidence in lung cancer (https://pmc.ncbi.nlm.nih.gov/articles/PMC4730048/), tumor suppressor evidence in ovarian cancer (10.31083/j.fbl2707210).

SPACA3	protein coding	ENSG00000141316	32,970,376	32,997,877	+	Sperm acrosome associated 3	Oncogenic evidence in colorectal cancer (10.1186/s12859-022-04657-3),
ASIC2	protein coding	ENSG00000108684	33,013,087	34,174,964	−	Acid sensing ion channel subunit 2	Oncogenic evidence in ovarian cancer (10.1038/s41598-025-03429-2), oncogenic evidence in colorectal cancer (10.1186/s13046-017-0599-9).
lncRNA antisense ASIC2	lncRNA	ENSG00000266535	33,111,853	33,152,748	+	Antisense to ASIC2	No data
